# Association of *TLR7* and *TSHR* copy number variation with Graves’ disease and Graves’ ophthalmopathy in Chinese population in Taiwan

**DOI:** 10.1186/1471-2415-14-15

**Published:** 2014-02-11

**Authors:** Wen-Ling Liao, Lei Wan, Tzu-Yuan Wang, Ching-Chu Chen, Siu-San Tse, Chieh-Hsiang Lu, Fuu-Jen Tsai

**Affiliations:** 1 Center for Personalized Medicine, China Medical University Hospital, No.2 Yuh-Der Road, 404 Taichung City, Taiwan; 2 Graduate Institute of Integrated Medicine, China Medical University, No.91 Hsueh-Shih Road, 404 Taichung City, Taiwan; 3 Department of Medical Genetics and Medical Research, China Medical University Hospital, No.2 Yuh-Der Road, 404 Taichung City, Taiwan; 4 School of Chinese Medicine, China Medical University, No.91 Hsueh-Shih Road, 404 Taichung City, Taiwan; 5 Department of Biotechnology, Asia University, No.500 Lioufeng Rd., 413 Taichung City, Taiwan; 6 Division of Endocrinology and Metabolism, Department of Medicine, China Medical University Hospital, No.2 Yuh-Der Road, 404 Taichung City, Taiwan; 7 Department of Surgery, Division of Urology, Tung’s Taichung Metroharbor hospital, No.699, Sec.1, Chungchi Rd., 435 Taichung City, Taiwan; 8 Division of Endocrinology and Metabolism of Internal Medicine, Ditmanson Medical Foundation Chia-Yi Christian Hospital, No. 539, 60002 Jhongsiao Rd., Chia-Yi City, Taiwan; 9 Department of Business Administration, National Chung Cheng University, No.168, Sec. 1, University Rd., Min-Hsiung Township, Chia-yi County 621, Taiwan; 10 TaTung Institute of Commerce and Technology, 253 Mi-Tuo Road, Chiayi City, Taiwan; 11 Department of Pediatrics, China Medical University Hospital, No.2 Yuh-Der Road, 404 Taichung City, Taiwan

**Keywords:** Graves’ disease, Graves’ ophthalmopathy, Copy number variation, TSHR, TLR7, Taiwan

## Abstract

**Background:**

Graves’ disease (GD) and Graves’ ophthalmopathy (GO) are autoimmune disorders, which might be influenced by genetic factors. Copy number variation (CNV) is an important source of genomic diversity in humans, and influences disease susceptibility. This study investigated the association between CNV in the *TSHR* and *TLR7* genes and the development of GD and GO in a Chinese population in Taiwan.

**Methods:**

For this case-control study, sample from 196 healthy controls and 484 GD patients, including 203 patients with GO were studied. CNV was detected by real-time polymerase chain reaction (PCR) using TaqMan™ probes and the relative copy number (CN) was estimated by using the comparative C_t_ method.

**Results:**

The differences in the distribution of *TSHR* CNV in healthy controls and GD patients were statistically significant (*p* value = 0.01). However, the difference in the distribution of *TSHR* CNV in the control group and the GO group was not statistically significant (*p* value = 0.06). For *TLR7* CNV, the results were not significantly different when we compared the distribution in healthy controls and GD patients and in healthy controls and GO patients (*p* values for Fisher’s exact test were 0.13 and 0.09, respectively). However, a lower than normal CNV for *TLR7* (CNV < 2 for female and CNV < 1 for male) was found to have a protective effect against the development of GD (odds ratio (OR) = 0.24; 95% confidence interval (CI), 0.07-0.75) after adjusting for age and gender.

**Conclusions:**

These results suggested that *TSHR* and *TLR7* CNV might be associated with susceptibility to GD.

## Background

Copy number variation (CNV) refers to is a genomic variation in the number of copies of a >1-kb DNA segment compared to a reference genome. Gene copy number (CN) differences due to deletions, duplications, and insertions might contribute to variations in gene expression, phenotypic variation, and gene dosage. Therefore, CNVs might play an important role in not only evolution and adaptation to different environments, but also in susceptibility to and severity of disease [1,2]. Recently CNV has been used as a genetic marker for inter-individual variation to investigate human genomic diversity. For example, the associations between CNV and diseases such as autism [3,4], schizophrenia [5], cancer [6-8], and autoimmune disorders [9-13] have been discussed in numerous studies.

Graves’ disease (GD) is an organ-specific autoimmune disease caused by autoantibodies against thyroid-stimulating hormone receptors (TSHRs); these antibodies constitutively stimulate the production of thyroid hormones. TSHR is a 7-transmembrane domain G protein-coupled receptor in the thyroid gland that binds thyroid-stimulating hormone (TSH). Therefore, the *TSHR* gene on chromosome 14q is a candidate disease-susceptibility gene for GD and the association between *TSHR* gene polymorphism and GD has been investigated [14-16].

Graves’ ophthalmopathy (GO) is the most common extrathyroidal manifestation of GD and affects 25–50% of patients with GD [17-20]. GO is an autoimmune inflammatory disorder that affects the extraocular muscles and the fatty and connective tissues in the orbit of the eye. Studies have shown that genes related to the immune system, such as *human leucocyte antigen (HLA)*, *cytotoxic T cell antigen-4 genes (CTLA), CD103* and *Interleukin13 (IL-13)* are involved in the pathogenesis of GD or GO [17,21,22]. Recently, toll-like receptors (TLRs), which play important roles in eliciting human innate/adaptive immune responses and developing chronic inflammation [23], have become a new focus of basic immunological investigation and might be associated with autoimmune thyroiditis [24]. *TLR7* gene, which is located on the X chromosome, is expressed in human plasmacytoid dendritic cells (pDCs) and specifically recognizes single-stranded RNA derived from viruses or immune complexes associated with self-RNA. This results in the activation of the pDCs and induces the secretion of large amounts of interferon alpha (IFN-α), which in turn has a pivotal role in the pathogenesis of GD/GO. Therefore, *TLR7* gene could be a potential immune-related disease-susceptibility gene for GD/GO. In this study, we examined the association of the CNV in *TSHR* and *TLR-7* with susceptibility to GD and GO in a population of Chinese people in Taiwan.

## Methods

### Study population

The participants in this study were 196 healthy controls and 484 GD patients which including 203 of them with ophthalmopathy (GO) recruited from China Medical University Hospital in Taiwan. All GD patients were examined by experienced endocrinologist and identified using following criteria: having hyperthyroidism, having diffused goiter and presence at least one of following observations: positive results for thyroid-stimulating hormone (TSH) receptor receptor antibody test, diffusely increased ^131^I (iodine-131) uptake in the thyroid gland, and exophthalmos. GD patients were categorized according the NOSPECS system recommended by the American Thyroid Association [25]. The GD patients had proptosis with or without more severe form (class 3-6) was defined as GO (yes/no). The degree of proptosis was measured using an exophthalmometer and was defined as the apex of the cornea and the lateral orbital rim is more than 18 mm in either eye or a 2-mm difference in the degree of protrusion between the 2 eyes. The information regarding age, sex, history of tobacco use, thyroid gland pathology and affected anatomic sites were obtained from the questionnaire and blood samples were collected by venipuncture for genomic DNA isolation. Informed consent was obtained from each participant prior to inclusion in this study and the human ethics committee of China Medical University Hospital gave its approval for the study.

### DNA preparation and TaqMan real-time polymerase chain reaction (rt-PCR)

The genomic DNA was extracted from peripheral blood leukocytes of participants using the Genomic DNA kit (Qiagen) according to the manufacturer’s instructions. It is important to verify the quality of each genomic DNA in order to get accurate CN. Therefore, all genomic DNA were electrophoresed on an agaroes gel and integrity scored from 1 to 4 (most degraded). Samples with scores of 3 and 4 were discarded from analysis. Also, in order to minimized DNA degradation, the template DNA were freshly diluted from 200 μg/ml stock and used within 2 weeks.

### Measurement of CN

The relative CN of the *TSHR* and *TLR7* gene in each individual was estimated using TaqMan real-time quantitative polymerase chain reaction (rt-PCR) method that is suited to large-scale genotyping. rt-PCR was performed using the ABI 7900 HT sequence detection system. Amplification reactions (14 μl) were carried out in duplicate with of template DNA (10 ng), reference DNA (RNase P H1 RNA gene (10 ng)), TaqMan Universal Master Mix buffer (Applied Biosystems, Foster City, CA), 300 nM of each primer and 200 nM of each fluorogenic probe (probe with FAMTM dye and VIC® dye). Thermal cycling was initiated with a first denaturation step of 10 min at 95°C, and followed by 40 cycles of 15 sec at 95°C and of 1 min at 60°C. The relative gene CN for each individual was estimated using the comparative Ct method (2–ΔΔCt method) [26]. Briefly, this method calculates the difference in cycle thresholds (ΔCt) (the number of PCR cycles required to produce a set of fixed thresholds) between the gene of interest and a reference gene (ribonuclease P, (RNase P). Subsequent calculations normalize the ΔCt of each sample to a calibrator that is assigned a relative expression value of 1.00 (ΔCt). Assuming that the amount of PCR product doubles with each successive PCR cycle, calculating the 2–ΔΔCt value will provide the relative amount of DNA initially available for amplification in each quantitative PCR run. Therefore, the 2–ΔΔCt method reveals differences in the relative gene copy numbers between the samples tested [27]. Here, RNase P which known to exist in two copies in a diploid genome is recommended as the standard reference assay for copy number quantitation experiments. This assay detects the RNase P RNA component H1 (H1RNA) gene (RPPH1) on chromosome 14, cytoband 14q11.2.

The PCR products were quantified in triplicate, and the standard deviation (SD) and coefficient of variation (CV) were calculated based on 3 runs. To control for reaction quality, each reaction plate also included a calibrator, a positive control, and a no-template control (NTC). Data from a plate were included if the calibrator CV was less than 5%, the positive control CV and sample CVs were all less than 10%, and the NTC was negative. To include across-plate data, the CVs of the positive control had to be similar and the NTC had to be negative.

### Data analyses

Significance of the differences between the distributions of *TSHR* and *TLR7* CNV in groups (healthy controls, GD and GO) was estimated by Chi square test or Fisher exact test. Odds ratios (OR) and 95% confidence intervals (CI) were estimated by logistic regression models. Age and gender were adjusted in multivariate models. Because the *TLR7* gene located on chromosome X, the interaction between gender and CNV of the *TLR7* gene was investigated. If the interaction term was not significant, males and females would be combined to further analyses. To estimate OR, the reference category was normal CNV which are CNV = 2 for *TSHR* gene, CNV = 2 for *TLR7* gene in females and CNV = 1 for *TLR7* gene in males. Based on the reference category, the risk of GD/GO associated with the absolute *TSHR/TLR7* CNV was estimated by comparing cases and controls groups by CNV less than normal CNV and CNV more than normal CNV.

## Results

In present study, the *TSHR* and *TLR7* genes showed variation in CN in a Chinese population in Taiwan. Specifically, 14.6% and 3.5% of this population had abnormal CNV for *TSHR* and *TLR7*, respectively.

### The distribution of *TSHR* copy number variation

The distribution of *TSHR* CNV in GD or GO patients is summarized in Table 1. There was association between *TSHR* CNV and GD (*p* value for Fisher’s exact test was 0.01). However, the distributions of *TSHR* CNV in the GO patients and healthy controls were not significantly different (*p* value for Fisher’s exact test was 0.06). In univariate analyses, a harmful effect of abnormal *TSHR* CNV (CNV <2) on the development of GD and GO were observed (OR = 2.63; 95% CI = 1.31-5.25 and OR = 2.35; 95% CI = 1.09-5.09 for comparing healthy control with GD and GO, respectively). After adjusting for age and gender, the results remain significant.

**Table 1:** **The distribution of *TSHR *** **copy number variation in healthy control and patients with Graves’ disease and Graves’ ophthalmology**

	**Healthy control (N = 196)**	**GD (N = 481)**	**GO (N = 203)**	**GD versus Healthy control**		**GO versus Healthy control**	
**CNV**	**N (%)**	**N (%)**	**N (%)**	**OR (95% CI)**^**b**^	**OR (95% CI)**^**c**^	**OR (95% CI)**^**b**^	**OR (95% CI)**^**c**^
**< 2**	10 (5.1)	59 (12.3)	23 (11.3)	2.63(1.31-5.25)*	2.61 (1.29-5.28)*	2.35 (1.09-5.09)*	2.54 (1.15-5.61)*
**2**	178 (90.8)	400 (83.2)	174 (85.7)	1	1	1	1
**>2**	8 (4.1)	22 (4.6)	6 (3.0)	1.22 (0.54-2.80)	1.34 (0.55-3.27)	0.77 (0.26-2.26)	0.98 (0.32-3.01)
***P value***^***a***^		0.01*	***0.06***				

GD, Graves’ disease. GO, Graves’ ophthalmology. CNV, copy number variance. N = number. OR, odds ratio. CI, confidence interval.

^a^*P* value was calculated by Fisher’s exact test. Statistically significant results are indicated by asterisk (*).

^b^Univariate analyses. Statistically significant results are indicated by asterisk (*).

^c^Mutivariates analyses, adjusted for age and gender, interaction term (gender*TSHR) was not significant. Statistically significant results are indicated by asterisk (*).

### The distribution of *TLR7* copy number variation

The distribution of *TLR7* CNV of healthy control subjects and GD patients is shown in Table 2. In healthy control subjects, 94.9% of healthy control and 97.1% of GD patients had normal *TLR7* CNV. The association between healthy control and GD or GO were not observed (*p* values for Fisher’s exact test were 0.13 and 0.09, respectively). When we adjusted age and gender in the multivariate logistic regression model, a protective effect of lower than normal CNV for *TLR7* (CNV < 2 for female and CNV < 1 for male), on development of GD was observed (OR = 0.24; 95% CI = 0.07-0.75). All of GD subjects with CNV less than normal CNV were females (n = 6). The interaction term between gender and CNV of the *TLR7* gene was investigated and the result was not significant (*p* value > 0.05).

**Table 2:** **The distribution of *TLR7 *** **copy number variation in healthy control and patients with Graves’ disease and Graves’ ophthalmology**

	**Healthy control (N = 196)**	**GD (N = 484)**	**GO (N = 203)**	**GD versus Healthy control**		**GO versus Healthy control**	
**CNV**	**N (%)**	**N (%)**	**N (%)**	**OR (95% CI)**^**c**^	**OR (95% CI)**^**d**^	**OR (95% CI)**^**c**^	**OR (95% CI)**^**d**^
**CNV < normal CNV**	7 (3.6)	6 (1.2)	1 (0.5)	0.34 (0.11-1.02)	0.24 (0.07-0.75)*	0.13(0.02-1.09)	0.13(0.02-1.05)
**Normal CNV**^**a**^	186 (94.9)	470 (97.1)	200 (98.5)	1	1	1	1
**CNV > normal CNV**	3 (1.5)	8 (1.7)	2 (1.0)	1.06 (0.28-4.02)	1.32 (0.33-5.26)	0.62 (0.10-3.75)	0.67 (0.11-4.15)
***P value***^***b***^		***0.13***	***0.09***				

GD, Graves’ disease. GO, Graves’ ophthalmology. CNV, copy number variation. N = number. OR, odds ratio. CI, confidence interval. #, number.

^a^CNV = 1 for male or CNV = 2 for female.

^b^*P* value was calculated by Fisher’s exact test.

^c^Univariate analyses. Statistically significant results are indicated by asterisk (*).

^d^Mutivariates analyses, adjusted for age and gender, interaction term (gender*TLR7) was not significant. Statistically significant results are indicated by asterisk (*).

## Discussion

In the present study, variation in the CNs of *TSHR* and *TLR7* gene was observed in the Chinese population in Taiwan, and the effects of this CNV on GD and GO were investigated. To the best of our knowledge, this is the first study to show that CNV in *TSHR* and *TLR7* is associated with GD/GO in the Chinese population in Taiwan. Previous studies on the effects of single nucleotide polymorphisms of the *TSHR* gene on GD have yielded inconsistent results [14,16,28]. Small study sample size and studying difference population for genetic diversity may be the reasons for these non-significant or inconsistent finding. But, a statistically significant association between CNV of the *TSHR* gene and GD/GO was identified in present study. Genetic variation within the *TSHR* region may influence *TSHR* structure, expression and/or post-translational processing, which in turn could initiate or exacerbate the autoimmune response against the *TSHR* in GD [29].

The *TLR7* gene belongs to a family of pattern-recognition receptors that recognize nuclear material from apoptotic debris. Researchers have demonstrated that *TLR7* plays an important role in producing IFN-α, which is a critical player in the innate and adaptive immune system [30,31]. Therefore, it is possible that CNV in *TLR7* gene could modulate the autoimmune response to nuclear material [12]. Our results showed that *TLR7* CNV is associated with genetic susceptibility to GD, with a wide confidence interval. The issue of limited study power due to the small effect size and limited sample size should be noted. Moreover, a link between *TLR7* gene dosage and increased autoimmunity has been identified in a mouse model of systemic lupus erythematosus (SLE) [32,33]; however, the effects of varying *TLR7* CN on SLE were inconsistent [10,12,33]. In the present study, a protective effect of lower than normal *TLR7* CNV (CNV = 1 for female) and a harmful effect of higher than normal CNV (result was not significant) on the development of GD was observed. However, a dosage effect of *TLR7* on GD/GO was not observed (data not shown). The relationship betweeen *TLR7* CNV, *TLR7* expression, and IFN-α activation will need to be investigated in future studies.

In our study, the effects of *TLR7* CNV on GD and GO were greater when the analyses were limited to female subjects. This gender-related difference in *TLR7* function has been observed previously. For example, Berghofer *et al*. (2006) showed that female SLE patients produce significantly more IFN-α after *TLR7* stimulation than male SLE patients. Epidemiological studies also indicate that GD predominantly affects women [34]. Many studies have confirmed that elevated levels of IFN-α play a key role in the development of clinical and experimental GD [35]. Therefore, *TLR7* CNV may contribute to the pathogenesis of GD in female patients through a gender-dependent immune response. However, the effect of *TLR7* CNV in male subjects is unclear owing to the small number of male subjects in this study. Therefore, a larger sample size, especially of the male population will be needed in future studies to confirm the importance of *TLR7* CNV as a genetic marker of GD.

## Conclusions

In conclusion, this study suggests that CNV in *TSHR* and *TLR7* is associated with the development of GD. *TSHR* and *TLR7* CNV might therefore be potential diagnostic tests or therapeutic targets for GD.

## Competing interests

The authors declare that they have no competing interests.

## Authors' contributions

WLL performed the statistical analysis and interpretation of data and drafted the manuscript. LW participated in the design of the study and carried out the molecular genetic studies. TYW and CCC participated in acquisition of data. FJT and CHL conceived of the study and participated in its design. All authors read and approved the final manuscript.

## Pre-publication history

The pre-publication history for this paper can be accessed here:

http://www.biomedcentral.com/1471-2415/14/15/prepub
